# 

**Published:** 1890-07

**Authors:** 


### SPECIAL.

We request that all our exchanges, correspondents and patrons, will take note of our change of location from 206 Broadway to 218 to 222 Fulton street, New York city, a necessity growing out of our greatly increased circulation and business.

We are now established in ample accommodations for the several departments immediately connected with the publication of our Journal, which we shall hereafter be able to bring out in better form, and with unfailing punctuality.

Once more we appeal to such of our patrons as are in arrears, to signify their appreciation of our endeavors to do them a service, by remitting their dues, before the publication of our forthcoming delinquent list. It is a small matter to them, individually, but to us, the aggregate of indebtedness on the part of subscribers, is a no inconsiderable barrier to our prosperity.

A word further respecting our advertising columns. It has been our purpose to confine these to the presentation of things of real merit, especially in regard to medicinal preparations and remedies for the prevention and cure of disease. This is an inflexible rule with us, and any infringement of it will be corrected on the first discovery. We concede that we owe this to our patrons, and they shall not be deceived, if we know it.

---

### ALCOHOLIC ADULTERATION.

BY S. H. PRESTON.

Alcohol in America is probably a poison in a more special sense than is popularly supposed. It is a chemically attested fact, but one only

### LITERARY.

NATIONAL HEALTH.—Abridged from the Health of Nations, of Sir E. Chadwick, K. C. B., by Benjamin W. Richardson, M. D., F. R. S. London, Longmans, Green & Co.—New York, 15 East 16th street. 1890. Price, $1.50.

This is one of the most valuable works of its kind ever published. The preliminary pages are given to a very comprehensive biographical sketch of the author of the more extensive work from which the present one was abridged, by his approval. Then follow a number of chapters upon the subject of Healthy Dwellings for Working People, and their construction, in detail, including water supply, drainage, and sanitary means and safeguards. Part II treats of "Health in the School," including the proper construction of school houses, school discipline, and the education of the masses, under just and reasonable regulations. Parts III and IV follow with a discussion of the "Health of the Community," wherein the temperance problem is considered at length, with suggestions for disencumbering society of its burdens by means of healthy and economical means.

This book will furnish the key to many much needed social reforms, for the bettering of individuals and communities, and should be in the hands of all whose calling is to conserve the public good.

CATALOGUE OF CLUBS.—We have received, with the compliments of P. F. McBreen, practical printer, 61 Beekman street, a copy of a book of 160 pages, of the above title, containing a list by name, date of organization and present officers, of some three hundred clubs in New York, Brooklyn, Jersey City and the near vicinity, formed for various objects.

This Catalogue is intended to be followed by annual revised editions. The illuminated copy before us is admirably gotten up, and reflects great credit upon its projectors. Club men will find it serviceable in many ways.

PAST, PRESENT AND FUTURE OF WOMAN, by Dr. Joseph Simms.—Dr. Simms has for a number of years devoted himself almost wholly to the study of the structural man—particularly his physiognomy, in nearly all sections of the globe.

The pamphlet before us of 40 pages, gives the preference to woman over her male companion, in almost every thing. The conclusion of the author is that "Woman is superior to man in every sphere where her culture has been equal to his." He is indeed very gallant, and we venture to say, that no true woman will deny his premises or dispute his conclusions.

---

CISTERNS.—After a summer drought all water tanks and cisterns should be examined, and, if need be, repaired, but in every instance well and carefully cleansed before being allowed to fill up again with water. Mischief is done, and disease induced and propagated, by the use of bad water, because the sediment, if not washed out, becomes mingled with every fresh influx of water. A very general and most virulent and fatal epidemic of diphtheria and severe attacks of typhoid fever have been known to be produced by the neglect of this essential duty. The necessity for frequent cleansing of cisterns cannot be too strongly insisted on.

SURVIVAL OF DANGEROUS GERMS.—It has been shown by M. Esmarch that disease microbes do not long survive in corpses, and that, as a general rule, the more rapidly decomposition takes place the more quickly will the organisms perish. Experiments were made with nine different kinds of microbes, contained in the bodies of animals under the various conditions of burial in the ground, keeping under water, and exposure to air. The bacillus of fowl cholera was seldom found after three weeks, though that of septicæmia survived 90 days, while that of consumption did not lose its virulence until from 204 to 252 days had passed. All trace of the other organisms—including those of typhoid fever, Asiatic cholera, tetanus and anthrax—disappeared in from three days to a week.

PLANT MOTION.—Motion seems to be a necessary endowment of life. "Plants," says Gray, "have no need of locomotion, and so are generally fixed fast to the spot where they grow. Yet many plants move their parts freely, sometimes when there is no occasion for it that we can understand, and sometimes accomplishing by it some useful end. The sudden closing of the leaflets of the Sensitive Plant, and the dropping of its leaf-stalk, when jarred, also the sudden starting forwards of the stamens of the Barberry at the touch, are familiar examples. Such cases seem at first view so strange, and so different from what we expect of a plant, that these plants are generally imagined to be endowed with a peculiar faculty denied to common vegetables. But a closer examination will show that plants generally share in this faculty; that similar movements may be detected in them all, only—like those of the hands of a clock, or of the shadow of a sun-dial—they are too slow for the motion to be directly seen."

---

### LILY WHITE.

When the summer days are fairest,
And the morn's caressing light,
Toys amid the blossoms rarest,—
Welcome be to Lily White.

Deftly now her mantle folding,
Through the dreamy hours of night;
Newly spread to morn's beholding,—
Gleeful, graceful Lily White.

Happy lays the wild birds sing her,
Born of freedom and delight,
Sweet the offerings they bring her,
Wooing, winsome Lily White.

Nature hath her loves approving,
Birds and flowers our pains requite,
But of all her ways of loving,
Thine are sweetest, Lily White.

LA CROIX.

